# Psychedelics: Their Limited Understanding and Future in the Treatment of Chronic Pain

**DOI:** 10.7759/cureus.28413

**Published:** 2022-08-25

**Authors:** Vedant N Hedau, Ashish P Anjankar

**Affiliations:** 1 Medicine, Jawaharlal Nehru Medical College, Datta Meghe Institute of Medical Sciences, Wardha, IND; 2 Biochemistry, Jawaharlal Nehru Medical College, Datta Meghe Institute of Medical Sciences, Wardha, IND

**Keywords:** psychological effects, serotonin receptor, drug trials, medicine, psychedelic drug research, chronic pain

## Abstract

Psychedelics are hallucinogenic drugs that alter the state of consciousness substantially. They bring about psychological, auditory, and visual changes. The psychedelics act on the brain, implying that they have a powerful psychological impact.

One of the main factors contributing to disability worldwide is pain. The majority of people deal with pain on a daily basis. Living with chronic pain affects daily life and has social implications. Chronic pain can be associated with any disease that may be genetic, idiopathic, or traumatic. The standard management of pain is done with pharmacological intervention and physical therapy. However, with time, patients may become resistant to a particular class of drugs. As these drugs do not help in treating the cause of pain, they act by blocking receptors and suppressing nervous systems, as this pharmacological intervention is not a permanent solution for pain management. Long-term use of the pharmacological intervention, which acts by suppressing the nervous system, may develop other side effects on the body. These standard therapies are not as effective in managing pain.

The opioid class of drugs has good pain-relieving properties but causes addiction; it needs therapeutic drug monitoring to monitor that it is not abused. Since the first synthetic psychedelic was developed, until today, we have had a fair chance to understand its effects and side effects.​These drugs are very potent and effective. They have shown promising developments in the field of clinical psychology. There is upcoming research on psychedelics' use in treating pain disorders.

In this article, let us understand the effect of psychedelic drugs on the brain and body and how they modulate pain. Even today, the precise mechanism of chronic pain is still not understood completely. Psychedelics' application and uses in future medicine and pain management are being studied. Understanding psychedelics' effects on the brain and how they function allows us to link how they might be used to treat chronic pain.

## Introduction and background

The term psychedelics originated from the Greek word for "mind manifesting" and was coined by Humphry Osmond, a British psychiatrist in Canada, in the 1950s [1]. Psychedelics are boon in psychiatry or a getaway to another realm. The most known drugs include LSD (lysergic acid diethylamide), DMT (dimethyltryptamine), mescaline, and psilocybin. LSD was produced in a lab in 1943 [2], while others are extracted from plants and fungi. Our ancestors likely ingested psychedelic mushrooms from the genus *Psilocybe *since the Pliocene (beginning 5.3 million years ago [mya]), when semi-arboreal hominins intensified foraging activity on the ground [3]. There is evidence of self-medication among the primates and Paleolithic humans [4]. Psychedelic substances hugely influenced certain cultures and religions dating back to 4000 BC [5].

Recent studies found that psychedelic-assisted therapy can effectively treat pain and mental health disorders [6]. Various reports of self-medication using psychedelics are documented, with anecdotal evidence of practical successes [7]. Suppose we consider our current pain medication regime, including non-steroidal anti-inflammatory drugs (NSAIDs), weak opioids, anti-depressants, and anticonvulsants. Most pain medication regime has various side effects if used for an extended period; for example, opioid use is linked chiefly with drug dependence and has contributed to the ongoing international opioid crisis [7]. Hence there are several problems with current treatments for pain management. Early studies in the 1960s and 1970s suggest that psychedelic drugs may help treat specific cancer pain and phantom limb pain [7]. The brain's activity under the influence of psychedelics affects the cerebral cortex, where sensory fibres terminate or motor fibres originate. Psychedelics have an "entropic" effect on neural connections [8]. They cause disruption, which leads to the formation of new nerve connections. Psychedelics may help in breaking down disorders by creating new connections. Many such theories and hypotheses require research and studies to understand underlying facts about how psychedelics affect the cerebral cortex and the body [9]. A recent survey of patients with chronic pain when self-medicated has shown a positive change in patients with chronic pain and toward the perspective of hope, empowerment, and optimism. Somatic presence fostered increased embodiment and was associated with prolonged analgesia [7]. Such studies in the field of psychedelics need to be researched and understood to bring effective changes in pain management.

Chronic pain is one of the leading causes of disability due to certain diseases and injuries. Currently, pharmacological interventions are ineffective in controlling pain, as they have many side effects, including sedation, dizziness, nausea, vomiting, constipation, physical dependence, tolerance, and respiratory depression [10]. Hence we need an alternative method for the treatment of pain. Psychedelics show promising results, as they are effective, non-addictive, and known side effects are rare. We need more understanding of these drugs on the human body and their effects on the brain, but the preliminary study done earlier in the 1950s - 1970s showed their positive effects [1]. Their impact on pain management in the future can be significant. Many people who self-medicate have reported drastic changes in their emotional or physical pain. Psychedelic drugs are potent and have long-lasting effects that may help people in their daily lives. Psychedelics use was associated with significant improvements in depressive and anxious symptoms and increased emotional wellbeing [11]. Evidence is available that psychedelic treatment can be beneficial for chronic pain. Patients on psychedelics experience disorientation and fail to recognize reality, have spiritual experiences and near-death experiences; patients cannot think or communicate normally. Considering all these short-term effects raises the question of their use, safety, and dosage administration [12]. These are the challenges that need to be considered in the study of these drugs.

## Review

Psychedelics: historical use and current approach

Psychedelics have been used pre-historically. Psilocybin, a type of fungi, has ancient origins. The earliest species dates back to a billion years ago. We know that indigenous people from Central America to Siberia used psychedelic mushrooms to bond with fellow humans and "higher powers" [13]. Evidence and texts suggest use of mischievous mushroom, spirits and magic potions in religious rituals. These mushrooms served as a conduit for them to communicate with their gods**.** In the Nahuatl language, which Mayan and Aztec people spoke, psilocybin was called "Teonanácatl", which translates to “flesh of the gods”. Ancient societies kept records of the Psilocybe mushrooms and their spiritual powers. In the 1500s, Spanish missionaries attempted to destroy all the evidence [13]. A 16th-century Spanish Franciscan friar, who moonlighted as a historian and shrooms enthusiast, mentioned Teonanácatl extensively [14]. His records intrigued 20th-century ethnopharmacologists and led to a decades-long search for the identity of Teonanácatl. The religious use of mushrooms by the Eurasian shaman is well documented [15]. Over the 2000 years that these rites were performed, a huge number of people experienced the impact of participating in this profoundly life-affirming and healing experience. During harvest celebrations, Eleusis' magical concoction known as Kykeon was frequently consumed to establish a connection with the grain god Demeter. Women, men, slaves, great emperors, and professors all used to partake in this preparation [16]. Similar to Mesoamerican societies, the ancient Egyptians left behind artworks depicting mushrooms. Our hominin ancestors invariably encountered and likely ingested psychedelics [3]. Hence, ancient history was influenced by psychedelics. 

Psychedelics again came to light in the mid-1960s and 1970s, in the era of artistic, musical, and social change. The word "psychedelics" is now frequently used to describe the multiple colours seen in drug-induced hallucinations. Many describe the experience of these drugs for creative problem-solving, recreational purposes, psychotherapy, and physical healing. In recent years, there has been tremendous interest in psychedelic research, with a growing number of conferences taking place in the world [17]. Breaking Convention, a biennial multidisciplinary conference on psychedelic awareness, is held in London, UK [18]. These drugs were extensively placed in Schedule I of the UN drug convention in 1967 [19]. Since 2006, several pilot trials and randomized controlled trials have used psychedelics (mostly psilocybin) in various non-psychotic psychiatric disorders. These have provided encouraging results that provide initial evidence of safety and efficacy; however, the regulatory and legal hurdles to licensing psychedelics as medicines are formidable [20].

Classification of psychedelics

Psychedelics mainly act on the 5-HT2a and dopaminergic receptor agonists [21]. They can be classified based on their structure (Table 1). They are grouped under tryptamines, phenethylamines, and lysergamides [22].

**Table 1:** Classification of Psychedelics Drugs [1, 23-25]

CLASS	DRUGS
1) Tryptamines	N, N-dimethyltryptamine (DMT), Etryptamine ; N,N-diethyltryptamine (DET), Psilocin, psilocybin
2) Phenethylamines	Mescaline, amphetamine, methamphetamine, 3-4 methylenedioxymethamphetamine (MDMA)
3) Lysergamides	Lysergic acid diethylamide (LSD), 1-propionyl-lysergic acid diethylamide (1P-LSD), 1-Acetyl-N,N-diethyllysergamide (ALD-52)

Psychedelics mechanism of action and activity in the brain 

The potent drug lysergic acid diethylamide's (LSD) psychoactive properties were discovered in 1943. The discovery of serotonin receptors occurred in 1953. The mental disturbances caused by LSD are attributed to inferences with serotonin receptors. 5-hydroxy-tryptamine (5-HT2A) has been considered the primary neurotransmitter system affected by psychedelics [21]. Additionally, LSD's hallucinogenic effect through binding with the 5-HT2A receptor can only occur upon dimerization with the metabotropic glutamate receptor 2 (mGlu2) [26]. Psilocybin increases striatal dopamine (DA) release [27]. In the presence of ketamine, pyramidal neurons in the prefrontal cortex exhibit increased frequency and amplitude of spontaneous excitatory postsynaptic potentials/currents (EPSPs/EPSCs) (layerV). Lower levels of lateral habenula (LHb) bursting activity in depressed animal models. Following cessation from amphetamines, the ventral tegmental region (VTA) had its DA neurotransmission restored.3-4 methylenedioxymethamphetamine (MDMA) increases long-term depression in the nucleus accumbens (NA). It decreases 5HT reuptake and decreases DA reuptake [28]. N, N-dimethyltryptamine(DMT) and ayahuasca increase the hippocampal gamma-aminobutyric acid (GABA), amygdalar GABA, amygdalar 5HT, DA, noradrenaline (NE), hippocampal 5HT, 5HT, DA and NE turnover with a decrease in amygdalar GABA [29]. So, the main site of action of psychedelic drugs are serotonin and dopaminergic receptors. Changes in these receptors impact cognition, perception, emotion, vision, and sense of body integrity [1]. People who have tried these drugs have experienced many effects, including visions and objects they have not seen before. Some describe this experience as an entry into a different dimension. Multiple visceral afferent and somatic signals are experienced over time, causing particular neural activity, causing sensitization resulting in physical and emotional pain experience [30].

Psychedelics' physiological action on the body

Psychedelics have effects on the serotonin receptors of the brain. Different classes of psychedelics produce different effects on the body. The most commonly used psychedelics and their effects are given in Table 2.

**Table 2:** Psychedelics and their action on the body [12,31]

Drugs	Physiological action
1) LSD ( lysergic acid diethylamide)	Increased heart rate and body temperature. Numbness, weakness, and tremors Impulsiveness and rapid emotional shifts that can range from fear to euphoria, with transitions so rapid that the user may seem to experience several emotions simultaneously, dizziness and sleeplessness, loss of appetite, dry mouth, and sweating.
2) DMT (N, N-dimethyltryptamine)	Increased heart rate, agitation, hallucinations, frequent and spatial distortions.
3) Ayahuasca	Increased blood pressure, severe vomiting (induced by the tea), profoundly altered state of awareness.
4) Psilocybin	Feelings of relaxation (similar to effects of low doses of marijuana), nervousness, paranoia, and panic reactions, introspective/spiritual experiences, misidentification of poisonous mushrooms.
5) MDMA (3-4 methylenedioxymethamphetamine)	Euphoria, a feeling of excitement, calm, or peace, feelings of well-being, heightened sensitivity, increased physical and emotional energy, increased sociability and closeness.
6) Mescaline	An altered state of consciousness. Dream-like state. Prominent changes in visual perceptions with intense visual distortions and possibly hallucinations. Development of vomiting, headaches, and feelings of anxiety.

Psychedelics are responsible for decrease in pain. In 1964, Kast, the Austrian-born physician, noted that the treatment given to underlying psychotic disease with a dose of LSD produces great results [32]. It offers a fast and effective way to enhance and understand the neurobiology of an altered state of mind. DMT reduces alpha beta bands and increases signaling activity [33]. Other same drugs in this category have a different mechanism of action on the basis of their structure, but collectively, it produces similar effects.

Psychedelics clinical study and trials

It is challenging for individuals in placebo-controlled trials who have never used psychedelic drugs. An original article published in 2021 conducted a study on 11 people who self-medicated with psychedelics drugs with the present treatment for pain [7]. An open-ended discussion was done with the participants by one of the researchers. The main takeaway of this original article was - how do people self-medicate with psychedelics; if it has any positive effects on the patient; and any suggestion or practice they feel would help in the medication. This information will help in developing future trials. The psychedelic drugs used have been efficiently understood due scientific advancements [34]. In a 1950s study, the outcome was positive. It showed they were effective in treating addiction and end-of-life distress [35]. These medications paved the path for molecular neuroscience research on their effects on the brain. Nonetheless, psychedelic substances were included to the UN's schedule I category [19]. However, still, the recreational use of psychedelics grew with time. The MDMA phase 3 trials have shown positive effects with a good safety profile [36]. Findings from nonclinical and clinical studies support a novel mechanism by which MDMA amplifies the therapeutic effects of psychotherapy through a dynamic interaction of brain regions and affiliated neurochemicals, known to be involved in fear extinction, learning, memory reconsolidation, emotional processing, and cognition [36]. Phase 2 clinical trial on the fibromyalgia patient shows that they have a significant therapeutic advantage over the prior medications in terms of their tolerability, effectiveness, and efficacy [7]. The procedure aims to relieve discomfort coming from the central nervous system. Standard treatment for fibromyalgia is ineffective and has many side effects. Patients with cancer who are suffering from emotional, psychological, and existential discomfort can be relieved by using the medication psilocybin, according to clinical trials.The result showed that (71-100%) of participants showed positive outcomes [37]. The participants rated the therapy as most essential and meaningful. Much clinical research in this period is going on in psychedelic drugs. More research and clinical trials are required to conclude its effectiveness in cancer pain treatment. The result is convincing, with fewer side effects. Hence, the future of psychedelics drug legalization looks good. Nevertheless, not every type of pain can be treated by psychedelics. Hence, conducting trials on selective diseases is necessary. We also need to understand these drugs' effective routes of administration. Traditionally, people took these drugs orally or through an inhalational route.

Psychedelics and their risk factors

There are short-term and long-term impacts associated with using psychedelics [38]. The individual action of psychedelics varies with drug and dose. In the short-term effects that last a few minutes to several hours, we observe the loss of sense of time, bright colours, and increased sensory and feeling experience. Long-term side effects involve disorganized thinking, paranoia, visual disturbance, and mood swings. If these drugs are administered in high doses, it can cause seizures, psychotic symptoms, amnesia, mood swings, and panic anxiety [38]. A disorder called hallucinogen persisting perception disorder (HPPD) can cause long-term effects [39]. In this, patients get flashbacks of previous drug use without any warning, causing distress and impairment. Nevertheless, even if administered in high doses, the effects of psychedelics are not life-threatening, but in severe overdose cases, they can cause coma or death [38]. These drugs generally do not cause addiction. They are better than the opioid class of drugs. However, LSD taken in large doses can cause the development of tolerance and may require more drug dosage to produce similar effects [40]. The uses of DMT are little known, but it does not appear to develop tolerance. More research is required in the field to understand the action and effects of these drugs, their usage, their dosage, and short- and long-term effects.

Future of psychedelic drugs in medicinal use

After psychedelics were listed in Schedule I of drugs by the UN, psychedelic research has come to light again in the last decade. Before drugs became outlawed, research carried out in the 1950s and 1970s showed promising preliminary results [41]. However, due to politics and the abuse of these drugs, they were declared illegal. Drug trials and research stopped, leaving us with a limited understanding of their effects. Many neuroscientists are interested in the molecular neurology of the serotonin receptor function of these drugs on the body. Advanced technologies are helping us understand their function well. Numerous neuroscientists have irrational theories regarding how psychedelic substances work ​[42]. In the coming years, we will be learning more about these drugs. Due to the widespread stigma associated with these medications, there are both legal and political obstacles to their introduction into mainstream medicine.

## Conclusions

Based on limited evidence from the past and recent studies on psychedelic drugs, we have a limited understanding of these drugs and their essential functions and mechanism of action. These drugs may not help cure all types of pain as the same drugs cannot cure all diseases. However, in chronic pain disorders, the patient’s life is impaired as they cannot perform daily functions. At present, pain management treatment is not ideal for patients. Psychedelic drugs may have the potential to be part of pain management treatments. We need more clinical studies and an understanding of the functions and actions of these drugs to bring them into use. We cannot ignore that psychedelic drugs were banned because of abuse. There is much social stigma around these drugs. Bringing them into clinical practice is a challenge. However, these therapies require more evidence and certification to be brought into daily practice. Nevertheless, the evidence and current studies going on in the field at the moment are promising. Hence, we will learn new facts in the coming time.
